# Physical, metabolic and developmental functions of the seed coat

**DOI:** 10.3389/fpls.2014.00510

**Published:** 2014-10-10

**Authors:** Volodymyr Radchuk, Ljudmilla Borisjuk

**Affiliations:** Heterosis, Molecular Genetics, Leibniz-Institut für Pflanzengenetik und KulturpflanzenforschungGatersleben, Germany

**Keywords:** seed development, nutrients supply, seed photosynthesis, PCD, maternal–filial interface

## Abstract

The conventional understanding of the role of the seed coat is that it provides a protective layer for the developing zygote. Recent data show that the picture is more nuanced. The seed coat certainly represents a first line of defense against adverse external factors, but it also acts as channel for transmitting environmental cues to the interior of the seed. The latter function primes the seed to adjust its metabolism in response to changes in its external environment. The purpose of this review is to provide the reader with a comprehensive view of the structure and functionality of the seed coat, and to expose its hidden interaction with both the endosperm and embryo. Any breeding and/or biotechnology intervention seeking to increase seed size or modify seed features will have to consider the implications on this tripartite interaction.

## INTRODUCTION

The evolution of sexual reproduction and the seed underlies much of the evolutionary success of the flowering plants. The most distinctive characteristic of the angiosperms is the double fertilization event, followed by the development of a seed encased in maternal tissue, referred to as the seed coat (or testa). The enclosure of the developing embryo affords it protection and thereby enhances its chances of reaching maturity and establishing the subsequent generation; this feature has not been achieved by species belonging to other clades of the plant kingdom. The progenitor structure of the angiosperm seed on the female side is the ovary, and its final form comprises an embryo, an endosperm, and the seed coat. The embryo results from the fusion between an egg cell and a sperm nucleus, while the endosperm develops from the fusion between the two central cell nuclei and a second sperm nucleus to produce (in diploid species) a triploid structure. The seed coat is entirely maternal in origin. When fertilization fails, the structure degenerates rapidly, thereby ensuring that the assimilate invested in an aborted seed is recycled (Roszak and Köhler, 2011). Post fertilization, the development of the seed relies on a coordinated interaction between the seed coat, the embryo, and the endosperm. The molecular basis of seed development has been intensively studied (Lafon-Placette and Köhler, 2014), but until now, the lack of suitable *in vivo* analytical methods has hampered systematic investigations of either the metabolism occurring or the internal structures developing within the growing seed. Here, a description is given of our current understanding of the functional role of the seed coat in the developing seed.

## FROM OVULE TO SEED COAT

The seed coat originates from cell layers surrounding the ovule. The analysis of a number of *Arabidopsis thaliana* mutants has revealed its structure and function, as well as identifying many of the genes involved in its development (Haughn and Chaudhury, 2005; Figueiredo and Köhler, 2014). Seed coat development is repressed prior to fertilization by dosage-sensitive, sporophytically active polycomb-type-proteins that are expressed in the maternal tissue surrounding the female gametophyte (Roszak and Köhler, 2011). The fertilization generates a signal that relieves the polycomb type protein-mediated repression, resulting in the initiation of seed coat formation (Roszak and Köhler, 2011).

**FIGURE 1:**
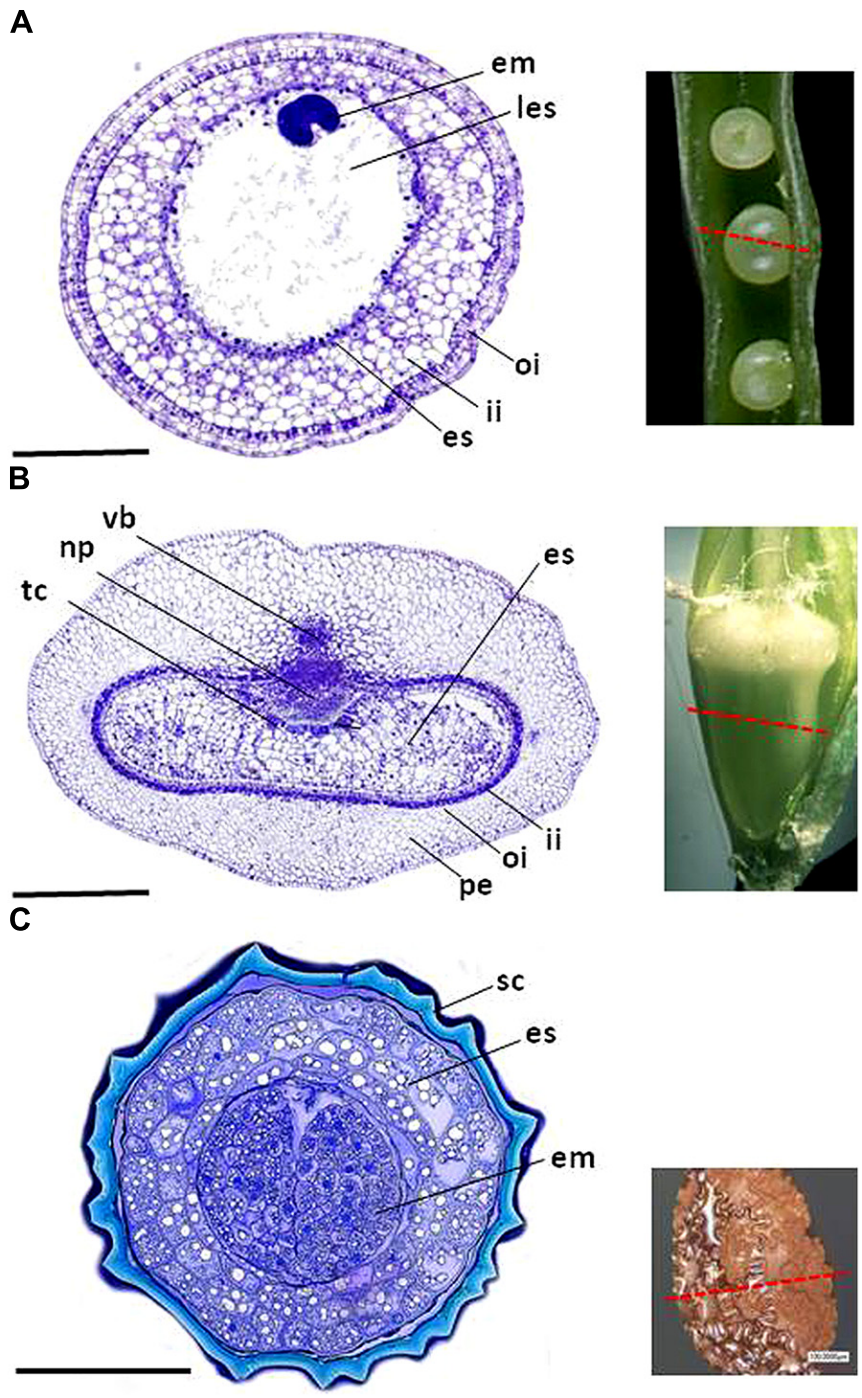
**The structure of **(A)** the oilseed rape seed, **(B)** the barley caryopsis, and **(C)** the tobacco seed.** em, embryo; es, endosperm; tc, endosperm transfer cell; ii, endothelium; les, liquid endosperm; vb, main vascular bundle; np, nucellar projection; oi, outer integument; pe, pericarp; sc, seed coat. Bars: 0.5 mm.

The *A. thaliana* seed coat is composed of five cell layers: the three-layered inner integument and the two-layered outer integument; each of these layers follows a distinct path during seed development. The endothelium (the innermost cell layer) synthesizes proanthocyanidins (PAs), which first condense into tannins, then oxidize to impart the brown pigmentation seen in the mature seed of many species (Lepiniec et al., 2006). The two adjacent cell layers are crushed together as the seed expands (Nakaune et al., 2005). The outer integument undergoes extensive differentiation, regulated by the *YABBY* family transcription factor *INNER NO OUTER* (Kelley and Gasser, 2009), going on to form the sub-epidermal and epidermal cell layers. The former of these generates a thickened wall on the side facing the epidermis (Haughn and Chaudhury, 2005), while the latter produces a pectinaceous carbohydrate referred to as mucilage (Arsovski et al., 2010; Haughn and Western, 2012). The outer integument is associated with a suberized layer, and the endothelium with a cutin-like polyester layer (Molina et al., 2008). In leguminous species, the seed coat is typically a multi-layered structure, including both macro- and osteosclerids in its outer integument and parenchyma in its inner integument (van Dongen et al., 2003; Verdier et al., 2012). In the cereal grain (strictly a caryopsis rather than a seed, since the ovary wall is fused with the seed coat), the endothelium and the outer integument each form a pair of cell layers, while the enlarged pericarp takes over some of the key functions of the seed coat (Sreenivasulu et al., 2010). The various impacts of the seed coat are illustrated for a contrasting set of species in **Figure 1**.

The development of the endothelium has been revealed by the analysis of *A. thaliana* mutants impaired in seed coat pigmentation. A number of relevant genes have been isolated, classified for the most part into either transcription factors or genes required for the synthesis and compartmentation of PA flavonoid compounds (Haughn and Chaudhury, 2005). Comprehensive transcriptomic descriptions of the developing *A. thaliana* seed coat have provided a wealth of information relevant to how the process occurs in other species (Dean et al., 2011). The *Medicago truncatula* myb transcription factor gene *MtPAR* has been shown to be a key regulator of PA synthesis, and its transcription co-localizes with the site of PA accumulation in the seed coat (Verdier et al., 2012). Key *M. truncatula* genes along with the precursor transporter *MATE1* (involved in PA synthesis) have been isolated and characterized by Zhao and Dixon (2009). Orthologs of *BANYULS,* which encodes anthocyanidin reductase (Albert et al., 1997), have been identified in oilseed rape and its close relatives *Brassica rapa* and *B. oleracea* (Auger et al., 2010). The transcriptional regulation of flavonoid metabolism is less well understood in legumes and cereals, perhaps because the genes underlying PA synthesis have been lost during domestication, with the result that white-seededness is commonplace in these taxa. Consequently, in contrast to its wild relatives, cultivated barley (similarly to rice and wheat) does not accumulate substantial amounts of PA (Sang, 2009). The relationship of secondary PA metabolism with both developmental regulation and the stress response has the potential to contribute significantly to future crop improvement and is being investigated by a number of research groups (Debeaujon et al., 2000; Bassoi and Flintham, 2005; Lepiniec et al., 2006; Gao et al., 2013).

Elucidation of the development of the *A. thaliana* outer integuments has relied on mutants that produce either less mucilage than the wild type or those which produce mucilage of a different composition. Both regulatory and structural genes have been recognized (Haughn and Chaudhury, 2005; Arsovski et al., 2010; Haughn and Western, 2012). The set of WD repeat, bHLH and *myb* transcription factors that regulate outer integument development partially overlaps with the factors controlling trichome initiation and development, the regulation of anthocyanin production and endothelial development, although the relevant interaction partners are distinct (Schiefelbein, 2003; Bernhardt et al., 2005; Haughn and Chaudhury, 2005; Gonzalez et al., 2008). For example, outer integument differentiation is controlled by the proteins TTG1, myb5/TT2, and TT8/EGL3, which also drive the transcription of *ABE1*, *ABE4*, *GH*, *GL2*, and *mybL2* (Gonzalez et al., 2008; Li et al., 2009). The *A. thaliana* model has been informative for understanding the molecular basis of the synthesis of cotton fibers, which arise from the epidermal cells of the outer integument and are distributed all over the seed’s surface (Lee et al., 2007; Liu et al., 2012; Ruan, 2013). Several of the regulatory genes involved in fiber initiation have proven to be homologs of *A. thaliana* genes (e.g., *TTG1* and *GL2*) responsible for trichome formation and the differentiation of the outer integument. The current understanding is that a transcriptional myb/bHLH/WD repeat complex is required for this initiation process (Yang and Ye, 2013). A full understanding of the regulatory machinery operating in the epidermal cells will aid in achieving further improvement in a number of cotton seed traits (Yan et al., 2009; Efe et al., 2010) as well as the development of sustainable means of processing seeds and the fibers (Kimmel and Day, 2001; Stiff and Haigler, 2012).

## NO LIFE WITHOUT PROTECTION

In many seeds, the epidermal layer of the seed coat generates a cuticle which represents a physical barrier between the seed and its external environment. Neither viruses nor bacteria are able to penetrate an intact mature seed cuticle (Singh and Mathur, 2004; Gergerich and Dolja, 2006). The only entry points into a mature seed of this type for a pathogen are the micropyle – which represents the point of entry of the pollen tube – and the funiculus, which links the maternal vascular system to the seed integument. The immature seed coat is less robust, so it offers less protection against pathogen penetration, which can occur via either the ovary wall or the stigma. Mechanically damaged cuticles offer an alternative path for pathogen invasion (Singh and Mathur, 2004). Integrity of seed coat surface is extremely important for seed quality and fitness during seed storage or germination, and diverse technologies are available for preserving and enhancing of seed surface (Black and Halmer, 2006; Brooker et al., 2007).

An additional layer of protection is provided in certain seeds by the deposition of toxic compounds such as cyanogenic glycosides, terpenoids, and flavonoids. The issue of seed coat chemistry has especial resonance in relation to the presence of glucosinolates in brassicaceous crops (Bohinc et al., 2012). The accumulation of phenolics in plant tissues is considered to be an adaptive response to adverse environmental conditions (Lattanzio et al., 2006; Vermerris and Nicholson, 2007).

Since plants lack mobile defender cells, they are forced to rely on the innate immunity of every cell and on the production of signal molecules by invaded cells and their subsequent sensing (Jones and Dangl, 2006). The small and highly stable cysteine-rich peptides referred to as defensins actively inhibit pathogen invasion in both plants and animals (Stotz et al., 2009). Defensins genes induced by pathogen infection have been identified in a number of plant species (Thomma et al., 2002; Lay and Anderson, 2005; Carvalho and Gomes, 2009). Their products are concentrated mainly in the peripheral/bordering cells, as typified in barley and rice (Kovalchuk et al., 2010), and are released following tissue damage (Thomma et al., 2002; Lay and Anderson, 2005). Defensin production can also be promoted by certain abiotic stress agents, and also by exposing plants to the phytohormones methyl jasmonate, ethylene, or salicylic acid (Lay and Anderson, 2005). The expression of defensins in response to a variety of biotic and abiotic stimuli implies the possibility of cross-talk between distinct signal transduction pathways and gene expression programs involved in cellular signaling and growth regulation (Hanks et al., 2005; Okuda et al., 2009). Plant defensins have become the focus of a considerable body of biotechnological research (Carvalho and Gomes, 2009; Kovalchuk et al., 2010).

**FIGURE 2:**
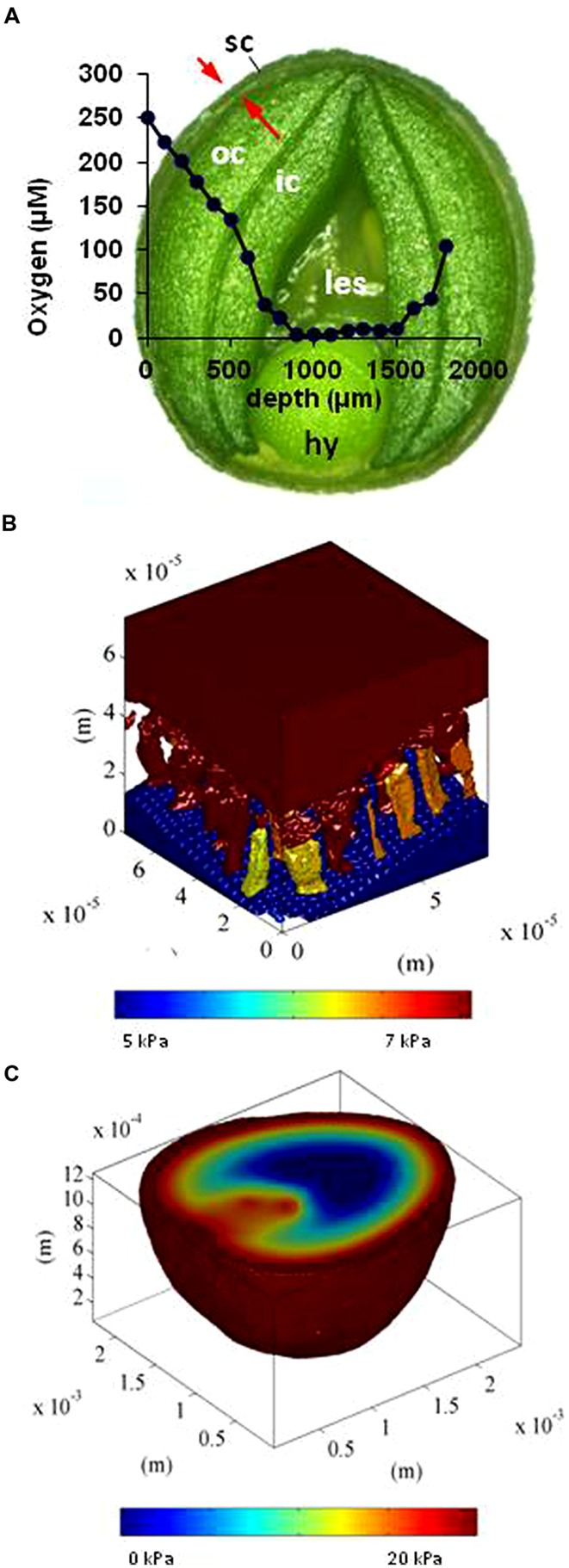
**Experimental measurements and *in silico* modeling of oxygen distribution in the developing oilseed rape seed. (A)** Oxygen concentration (blue line) along a transect of the seed, as determined by a needle micro-sensor. The x axis plots the penetration of the sensor. **(B)** *In silico* modeling of oxygen concentration in the pore spaces of the seed coat [indicated by arrows in **(A)**]. **(C)** Oxygen concentration map (color-coded). For details see Verboven et al. (2013). hy, hypocotyl; ic, inner cotyledon; les, liquid endosperm; oc, outer cotyledon; sc, seed coat.

The barrier function of the seed coat does not extend to gases, since it is in most cases at least semi-permeable (Welbaum and Bradford, 1990; Beresniewicz et al., 1995). The seed coat epidermis in the mature seed features no, or at best only scarce, functional stomata (Cochrane and Duffus, 1979; Geisler and Sack, 2002). In conjunction with the chemical composition of the cuticle, this implies a rather limited capacity for gas exchange (Nutbeam and Duffus, 1978; Sinclair et al., 1987; Sinclair, 1988). It was already demonstrated some 40 years ago that most of the gas exchange activity occurring within the pea seed is located in the micropylar region (Wager, 1974). The diffusivity of carbon dioxide through plant tissue is much higher than that of oxygen, since (unlike oxygen) carbon dioxide is readily soluble in water and so can move from cell to cell in the form of the carbonate ion. The presence of gas-filled intercellular spaces is therefore likely to be essential for translocation of oxygen within the seed. Synchrotron X-ray computer tomography has identified such spaces in the developing seeds of both *A. thaliana* (Cloetens et al., 2006) and oilseed rape *in vivo* (Verboven et al., 2013). In the latter species, both the seed coat and the hypocotyl are well supplied with void spaces, unlike the cotyledons, where the spaces are small and only poorly inter-connected (**Figure 2**). *In silico* modeling has revealed a three orders of magnitude range in oxygen diffusivity from the seed coat to particular embryonic tissues (Verboven et al., 2013). The multiple void spaces present in the seed coat suggest that gas exchange is effective within this part of the seed. There is a lack of any interconnectivity with the embryo, so the seed coat void network is likely to be autonomous. Both the seed cuticle and the lipid-containing aleurone layer of the endosperm have been identified as barriers to oxygen exchange, the former between the seed coat and external atmosphere and the latter between the seed coat and the endosperm/embryo. The oxygen pool stored in the voids of oilseed rape seed is consumed about once per minute. Since the developing seed has a high respiratory rate, it requires an additional supply of oxygen to maintain aerobic respiration.

Oxygen micro-sensor measurements made within the seed of faba bean, pea (Rolletschek et al., 2002, 2003) and soybean (Borisjuk et al., 2005), and within the grains of barley (Rolletschek et al., 2004), wheat (van Dongen et al., 2004), and maize (Rolletschek et al., 2005) have established that hypoxia is the norm. In the developing seed, it may be advantageous to keep the oxygen level low, because the bioenergetic efficiency of mitochondria is usually increased at low oxygen levels (Gnaiger et al., 2000). Thus, a low internal oxygen concentration in the seed may stimulate carbon use efficiency. Low oxygen levels help to avoid the formation of toxic concentrations of reactive oxygen species, which damage cellular structures and require the expenditure of energy for repair (Borisjuk and Rolletschek, 2009). In maize, the level of expression of detoxification genes (encoding glutathione *S*-transferase, superoxide dismutase, and ascorbate peroxidase) decreases during grain development (Méchin et al., 2007), consistent with a reduction in oxygen availability. To summarize, maintaining a low oxygen level within the seed has been proposed to provide a means for the developing seed to control the local level of metabolic activity (Borisjuk and Rolletschek, 2009). Deep within the mature seed, the inhibition of gas exchange can generate a state of near-anoxia, which may help to ensure the remarkable longevity of seeds (Shen-Miller, 2002). While the mechanistic basis of seed longevity is not fully understood, an important component is likely the control of oxidation (Hendry, 1993; Smirnoff, 2010; Bailly and Kranner, 2011). Practical methods to prolong seed viability in *ex situ* gene banks exploit this natural phenomenon by hermetically sealing the seed in order to maintain a high level of carbon dioxide within; this is combined with careful drying down and refrigeration, which help to slow seed metabolism/respiration and suppress oxidation processes (Kranner et al., 2010).

## PERCEIVING ENVIRONMENTAL CUES

The seed coat’s function is simultaneously to protect the embryo and to transmit information regarding the external environment. An impenetrable seed coat may help to keep the embryo safe, but at the same time it would exclude the sensing of environmental cues. The evolutionary solution to this dilemma is to combine certain structural features with appropriate levels of metabolic and photosynthetic activity in the seed coat.

**FIGURE 3:**
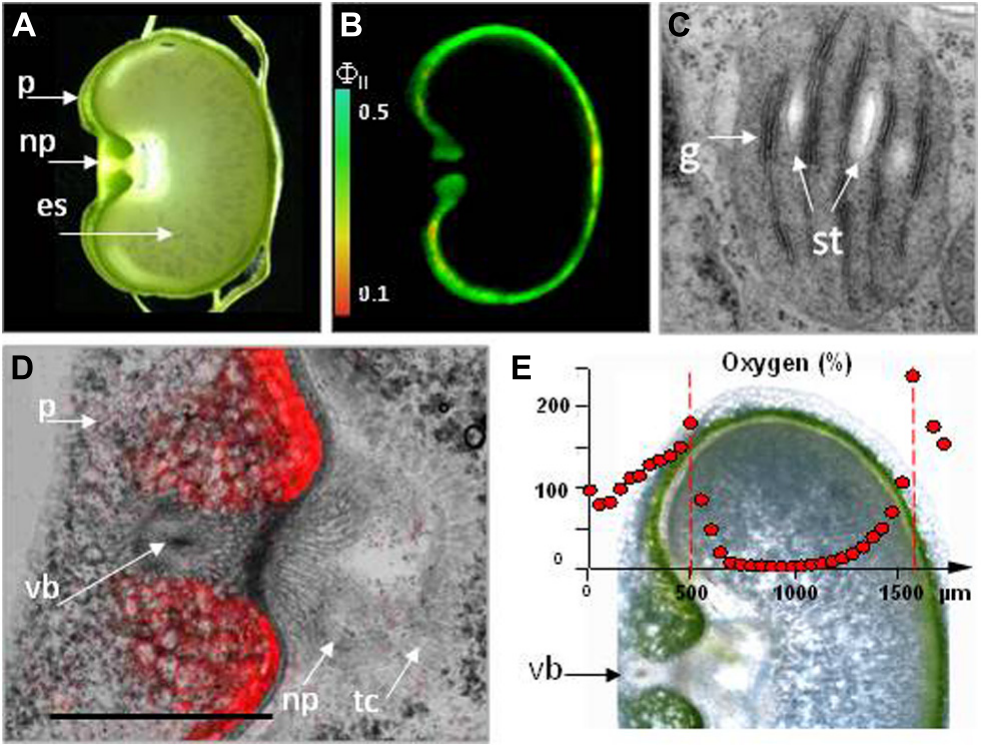
**Photosynthesis in the barley pericarp. (A)** Cross-section of a grain. **(B)** The effective quantum yield of photosystem II (FII) across a cross-section of a 12-day-old caryopsis, measured at a light intensity of 160 μmol quanta m^-2^ s^-1^. The scale shows the relationship between the color and FII. **(C)** A transmission electron micrograph of seed chlorenchyma plastids. **(D)** Chlorophyll auto-fluorescence within the crease region of the pericarp. **(E)** Oxygen levels within the caryopsis, as measured by a micro-sensor. For details see Tschiersch et al. (2012). es, endosperm; g, grana; np, nucellar projection; p, pericarp; st, starch grain; tc, transfer cell; vb, vascular bundle.

As plant species vary so much with respect to the distribution and amount of chlorenchyma in their developing seed, it is difficult to make meaningful generalizations regarding seed photosynthesis. However, out of 19 major crop species, only maize grains lack chlorophyll (Bewley and Black, 1994). Both the seed coat and embryo of pea (Tschiersch et al., 2012), soybean (Saito et al., 1989), oilseed rape (Borisjuk et al., 2013) and faba bean (Rolletschek et al., 2003) are photosynthetically active during seed development. The immature caryopsis of barley, wheat, rice and other grasses features a photosynthetically active pericarp (Bewley and Black, 1994; Rolletschek et al., 2004). The site of photosynthetic electron transport coincides with that of chlorophyll (Tschiersch et al., 2012), as for example in the barley pericarp (**Figures 3A,B**). When exposed to light, the chloroplastids (**Figure 3C**) produce sufficient ATP and NAPDH to meet local energy demand. Given the very short half life of both ATP and NADPH, it is likely that little long distance transport occurs from their site of synthesis. Non-photosynthetic plastids within the pericarp depend entirely on an external supply of ATP, just as is the case for other non-photosynthetic tissues (Möhlmann et al., 1994; Möhlmann and Neuhaus, 1997). The spatial separation between the endosperm and the photosynthetically active pericarp implies that seed photosynthesis does not make any direct energy contribution to assimilate storage in the endosperm.

Photosynthetic activity in the seed coat, as in the leaves, fixes carbon dioxide (Nutbeam and Duffus, 1978; Caley et al., 1990), and generates oxygen (Rolletschek et al., 2004; Tschiersch et al., 2012). This process is saturated in seeds at a light intensity some fivefold below that applicable for leaves. Oxygen production and carbon dioxide fixation combine to maintain a consistent gaseous environment within the seed. The effect of photosynthetic oxygen evolution exceeding the oxygen demand of the respiring seed coat is an increase in the internal oxygen level (Patrick et al., 1995; Rolletschek et al., 2002, 2003), which serves to relieve hypoxic stress and thereby enhances the synthetic activity of the seed (Greenway and Gibbs, 2003; Rolletschek et al., 2005). Importantly, the tissues through which nutrients are transported to the endosperm/embryo are oxygen depleted (Melkus et al., 2011). In the dark, the oxygen level can fall below 0.1% of the ambient atmospheric concentration (Rolletschek et al., 2011), but the chlorenchyma layer that surrounds the region ensures that the level of oxygen present is much higher in the light than this (**Figures 3D,E**). Both nutrient transport to, and storage activity within the endosperm rely heavily on respiratory energy and thus on a steady supply of photosynthetically derived oxygen. Experiments tracking the incorporation of labeled sucrose into starch have shown that the process is stimulated by both light and oxygen (Gifford and Bremner, 1981), underlining the dependence of storage activity on a supply of oxygen. Similarly, assimilate supply to the dicotyledonous seed is also oxygen-dependent, as shown by phloem unloading experiments (Thorne, 1982).

The seed coat’s high rate of respiration, along with its low permeability with respect to carbon dioxide, contributes to elevating the seed’s internal level of carbon dioxide. However, high concentrations of carbon dioxide do promote phosphoenolpyruvate carboxylase activity, which serves to encourage carbon dioxide re-fixation and so restricts its loss (Wager, 1974; Harvey et al., 1976; Flinn, 1985; Araus et al., 1993; Golombek et al., 1998). Limiting carbon dioxide loss in this way can make an important contribution to the seed’s overall carbon budget (Vigeolas et al., 2003; Rolletschek et al., 2004). In addition, re-fixation of carbon dioxide is mediated by Rubisco activity during photosynthesis (Goffman et al., 2004; Ruuska et al., 2004). The seed’s rate of carbon dioxide uptake from the atmosphere is much lower than the leaf’s (Brar and Thies, 1977; Watson and Duffus, 1988), in line with both a lower activity of photosynthesis-associated enzymes (Duffus and Rosie, 1973) and a limited rate of metabolic turn-over (Schwender and Ohlrogge, 2002; Sriram et al., 2004). Critical factors should be considered such as (1) the low density of stomata on the surface of the developing seed, and (2) the low amount of chlorenchyma. Most relevant experiments have disregarded the re-assimilation of internally produced CO_2_ and hence probably underestimated the actual sizes of the occurring fluxes (Araus et al., 1993). Nevertheless, the generally held conclusion still stands that the contribution of seed photosynthesis to dry matter production (via net CO_2_ fixation) is low.

One likely hypothesis regarding the evolutionary significance of retaining photosynthetic capacity in the seed coat suggests that the interception and processing of light by the seed coat gives the seed the means to sense its external environment, which is integrated with the hormonal, metabolic, and other signals brought to the seed through the phloem. Assimilate generated in the leaf is exported into the phloem in the form of sugar. The developing seed can benefit from the capacity to anticipate a burst of sugar arriving via the phloem, since this would facilitate the seed’s rapid adjustment to its synthesis of storage products. Photosynthesis has a marked effect on the entrainment and maintenance of robust circadian rhythms (Haydon et al., 2013). In this way, the retention of seed photosynthesis can provide a means of tuning the seed’s metabolism to the quantity and quality of the light available to the mother plant.

## HIGHWAYS AND BYWAYS TRAVELED DURING SOLUTE TRANSFER

**FIGURE 4:**
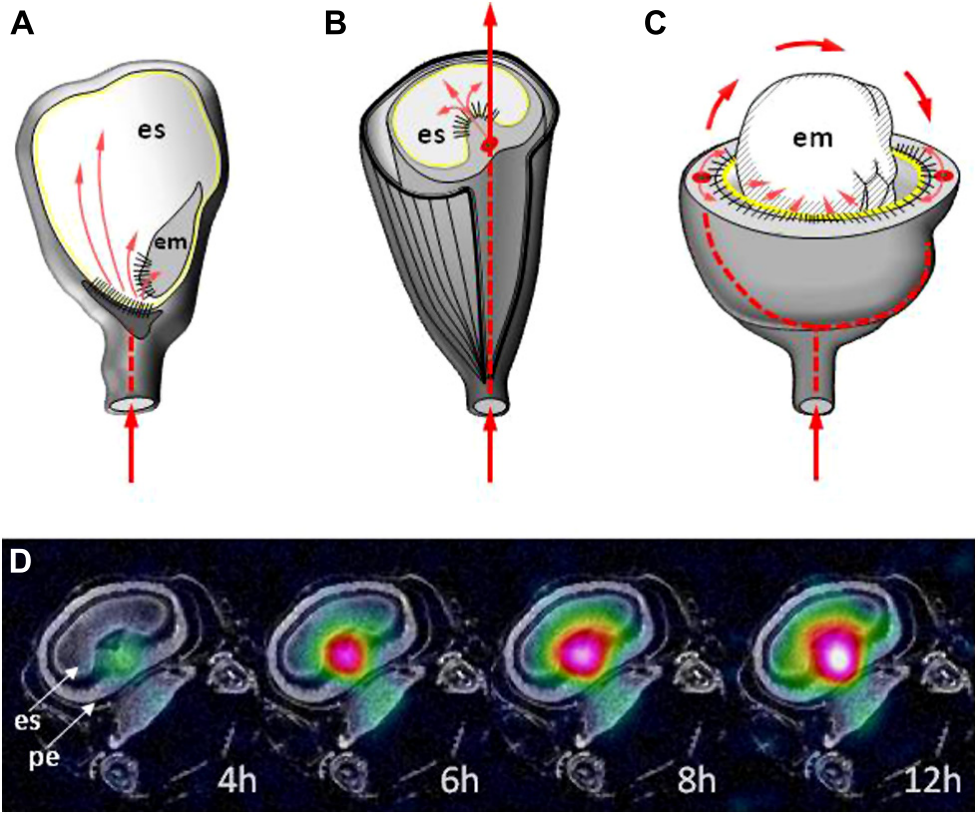
**The vascular bundle (thick red lines) and post phloem routes (thin red arrows) of solute transfer in **(A)** maize, **(B)** barley, and **(C)** pea.** The yellow line indicates the outer surface of the endosperm and the black stripes indicate the maternal–filial interface. **(D)** The monitoring of sucrose allocation (indicated by color code) resulting from a 12-hour period of feeding with 13C sucrose to the stem at the onset of seed filling stage in barley. The time elapsed since the beginning of the feeding is shown. For details see Melkus et al. (2011). em, embryo; es, endosperm; pe, pericarp.

Assimilates produced by the mother plant are delivered to the developing seed via the same conduit, the vascular system, which brings hormonal signals and the necessary protein- and RNA-based messages. Collectively, this enables the coordination of physiological and developmental processes at the whole organism level (van Bel et al., 2013). As the vascular system does not extend beyond the seed coat (Patrick et al., 1995), the embryo and the endosperm are apoplastically isolated from the mother plant and are therefore somewhat autonomous. Several pathways for nutrient flow are available, depending on seed size and structure. The smallest seeds (orchid seeds can be as small as 200 μm in diameter) have no vascular structure; rather, the zygote forms a haustorium which extends toward the terminus of the mother plant’s vascular system. Slightly larger seeds form a bundle of pro-vascular elements. Medium-sized seeds develop a simple, well developed collateral bundle (van Dongen et al., 2003). Finally, in large-seeded species, the vascular bundle is bulky enough to anastomose, thereby allowing for the distribution of nutrients throughout the seed (Vinogradova and Falaleev, 2012). In the *A. thaliana* seed, the vascular tissue terminates at the junction of the funiculus and ovule, and in the maize kernel, the vascular bundle terminates at the placenta–chalazal region (**Figure 4A**; Dermastia et al., 2009; Gómez et al., 2009; Costa et al., 2012). In contrast, wheat, barley and rice grains form a vascular system that extends over the whole length of the grain (**Figure 4B**; Sreenivasulu et al., 2010). The vascular architecture of *Fabaceae* species seeds is highly variable, ranging from a single chalazal vein in the *Viciae* and *Trifolieae* to an extensive anastomosed arrangement in the *Phaseoleae* (**Figure 4C**; van Dongen et al., 2003; Weber et al., 2005; Verdier et al., 2013).

Vascular structures are typically embedded within parenchymatous tissue. Adjacent parenchyma cells are interconnected by plasmodesmata, forming a symplastic continuum (domain). The plasmodesmata within these domains are larger than elsewhere in the seed (Ruan and Patrick, 1995; Oparka et al., 1999; Stadler et al., 2005a,b), which facilitates the movement of small molecules such as sugars and peptides. The maternal symplasm represents the major route for nutrients to reach the seed (Wang and Fisher, 1994; Borisjuk et al., 2002; van Dongen et al., 2003). In *A. thaliana*, each integument forms an independent symplasm (Stadler et al., 2005a; Ingram, 2010), which acts as an extension of the phloem (Stadler et al., 2005a,b). In common bean, faba bean, and pea, assimilate unloading sites are distributed throughout the seed coat parenchyma, with the possible exception of the branched parenchyma (Patrick and Oﬄer, 1995). In the small grain crops, the nucellar projection is the focus of a well organized transport route. The cellular architecture of the nucellar projection has been described in wheat (Wang and Fisher, 1994) and barley (Thiel et al., 2008; Melkus et al., 2011). A characteristic feature of tissues adjacent to the nutrient transport route is the presence of multiple symplastic junctions, large intercellular spaces and cell wall invaginations. The cells of the nucellar projection are extended toward the endosperm, thereby directing the flow of nutrients into the seed. With the exception of the crease region, a thick cuticular layer borders the pericarp and encloses the whole endosperm. In rice, two routes have been identified for nutrients to reach the developing grain: one is analogous to the nucellar projection, while the second passes through the nucellar epidermis (Oparka and Gates, 1981; Krishnan and Dayanandan, 2003).

The structure of the seed impedes the direct visualization of the site of the interaction between maternal and filial tissue. Various dyes and fluorescence- or isotope-labeled substances have been employed to follow nutrient (mainly sucrose) transport (Fisher and Cash-Clark, 2000; Stadler et al., 2005a,b). However, this experimental approach has the major disadvantage of being destructive. Invasive methods inevitably risk inducing artifacts with respect to both metabolite distribution and enzymatic activity. Non-invasive technologies, in the form of biosensors or imaging platforms like Foerster resonance energy transfer (FRET), Positron Emissions Tomographie (PET), and nuclear magnetic resonance (NMR) provide potentially superior alternatives (Frommer et al., 2009; Jahnke et al., 2009; Borisjuk et al., 2012). Real-time information on signaling and metabolite levels with subcellular granularity can be obtained *in vivo* with the help of genetically encoded FRET nanosensors (Frommer et al., 2009). PET does appear to be an appropriate platform for *in planta* analysis (Jahnke et al., 2009). When ^11^C is the target isotope, its spatial resolution of 1.4 mm (Phelps, 2004) suits it for the study of long distance translocation. NMR – and especially ^13^C NMR – is less sensitive than PET, but it delivers a fivefold higher level of in-plane resolution than PET, and can be used for real time monitoring (Melkus et al., 2011). The dynamic NMR-based imaging of sucrose in barley seed was integrated with flux balance analysis (FBA), which operated with more than 250 biochemical and transport reactions occurring in the cytosol, mitochondrium, plastid, and extracellular space. This approach has helped to unravel the complex biochemical processes affecting sucrose distribution in the grain (Melkus et al., 2011; Rolletschek et al., 2011).

## DELIVERING NUTRIENTS ACROSS THE MATERNAL–FILIAL INTERFACE

Some experimental evidence has been obtained to support the view that the delivery of metabolites to the embryo bypasses the endosperm (Yeung and Meinke, 1993; Weijers et al., 2003; Stadler et al., 2005a; Morley-Smith et al., 2008; Ungru et al., 2008; Pignocchi et al., 2009). However, other data suggest that the process is, in fact, mediated by the endosperm, largely because compromised endosperm development is so often associated with aberrant embryo growth (Chaudhury et al., 2001; Choi et al., 2002; Garcia et al., 2005; Ingouff et al., 2006). In either case, the inter-generational transfer of materials occurs via the apoplastic space. The specialized cellular structures developed at the tissue margins coordinate nutrient delivery from the seed coat into the seed itself. Transfer cells develop invaginated cell walls, thereby increasing the surface area of their plasma membrane and hence their capacity to transport nutrients (Andriunas et al., 2013). Nutrient transporters such as sucrose transporter 1 (Weber et al., 2005; Melkus et al., 2009) and amino acid permease 1 (AAP1; Tegeder, 2014) are typically present in both maternal and filial cells. Often they appear as tissue-specific isoforms: examples are the tonoplast intristic proteins (TIPs) for water (Gattolin et al., 2010) and Siliques Are Red 1 (SIAR1) for amino acids (Ladwig et al., 2012); some can change from eﬄux to influx mode in response to metabolic signals (Ladwig et al., 2012). The transfer cells positioned on either side of the apoplast (Zhang et al., 2007) act as the gateway for nutrient flow, as demonstrated *in vivo* by NMR in the barley caryopsis (**Figure 4D**; Melkus et al., 2011; Rolletschek et al., 2011).

The maternally located eﬄux transfer cells are responsible for the release of nutrients into the apoplast, and form cell wall ingrowths which direct the flow toward the seed (Stadler et al., 2005b; Zhang et al., 2007). The plasma membranes in these cells are enriched with respect to aquaporins, membrane transporters, and channels for sugars, amino acids and peptides, inorganic ions, and other compounds (Zhang et al., 2007; Thiel et al., 2008; Bihmidine et al., 2013). These eﬄux cells, like the cells of the barley and wheat nucellar projections, typically undergo programmed cell death (PCD; Zhou et al., 2009; Radchuk et al., 2011), which contributes to nutrient transfer to the filial tissue. In the maize seed coat placenta–chalazal region, PCD is coordinated with endosperm cellularization and is completed prior to the beginning of the storage phase. In this way, PCD functions as an adaptive process to facilitate the passage of solutes (Kladnik et al., 2004). In barley, the extensive vacuolization of cells in the nucellar projection allows for the transient accumulation of sucrose, which is released together with the complete cell contents to the apoplast after cell disintegration. A defective nucellar projection compromises nutrient flow into the endosperm, resulting in a reduction in final grain size (Radchuk et al., 2006; Melkus et al., 2011; Yin and Xue, 2012). Although important for the seed’s fate, the identity, and mechanics of eﬄux constituents and transporters are only poorly understood (Braun, 2012; Patrick et al., 2013).

The influx transfer cells in monocotyledonous species lie on the surface of the endosperm, directly opposite the maternal unloading site (Thiel et al., 2008; Monjardino et al., 2013; Lopato et al., 2014). The development and function of these transfer cells have been comprehensively and recently reviewed (Lopato et al., 2014; Thiel, 2014). In dicotyledonous species, the transfer cells usually face the seed coat (Borisjuk et al., 2002; Oﬄer et al., 2003; Olsen, 2004). A delay in the *trans*-differentiation of the embryonic epidermal cells to form transfer cells in the pea mutant *E2748* has a negative impact on embryo growth and seed viability (Borisjuk et al., 2002). Maize grains defective for the formation of basal endosperm transfer cells exhibit a shrunken kernel phenotype, as exhibited in the mutants *reduced grain filling 1* (Maitz et al., 2000), *globby1* (Costa et al., 2003), *baseless1* (Gutiérrez-Marcos et al., 2006), *empty pericarp 4* (Gutiérrez-Marcos et al., 2007), and *miniature1* (Kang et al., 2009).

The coordinated differentiation of opposing transfer cells requires a functional interaction between them, so presumably relies on an effective signaling mechanism. How this interaction operates is unclear, but a possible sequence of events has been suggested by Weber et al. (2005) and Andriunas et al. (2013). In the dicotyledonous seed, the expanding cotyledon makes contact with the seed coat, after which the innermost thin-walled parenchyma cells are gradually crushed (Oﬄer et al., 1989; Harrington et al., 1997). The stress, akin to wounding, may induce an ethylene burst (Harrington et al., 1997; Zhou et al., 2010). In response, a secondary ethylene burst in the adjacent embryo cells could be mediated by the auto-regulated expression of 1-amino-cyclopropane-1-carboxylic (ACC) synthase (Chang et al., 2008). The process initiates the *trans*-differentiation of epidermal cells into transfer cells (Zhou et al., 2010). Crushing of the seed coat is also coupled with a decrease in the activity of extracellular seed coat-specific invertase (Weber et al., 1996a,b), which leads to a local reduction in the level of intracellular glucose (Borisjuk et al., 1998). The lowered glucose level, sensed via a hexokinase-dependent pathway, removes the glucose-induced repression of ethylene-insensitive 3 (EIN3) and triggers an ethylene-signaling cascade, driving transfer cell differentiation (Dibley et al., 2009; Andriunas et al., 2011). As shown in both *in vitro* and *in vivo* experiments, transfer cell formation across a wide range of plant species involves an interaction between phytohormones, sugar, and reactive oxygen species (Dibley et al., 2009; Forestan et al., 2010; Zhou et al., 2010; Andriunas et al., 2012; Xia et al., 2012). The signals and signaling pathways responsible for the induction of transfer cell formation may be conserved across the monocotyledon/dicotyledon divide (Andriunas et al., 2013). When the promoter of the maize transfer cell-specific transcription factor *ZmMRP1* (Gómez et al., 2009) was fused to a *GUS* reporter gene and inserted into maize, *A. thaliana*, tobacco, and barley, GUS activity could be identified in regions of active transport between source and sink tissues in each of these species (Barrero et al., 2009), supporting the idea that the processes involved in transfer cell differentiation are similar across a diversity of plant species, and that differentiation is initiated by conserved induction signals.

## COMMUNICATING BETWEEN ADJACENT SEED COMPARTMENTS

Coordination of seed development clearly requires communication between seed compartments, and in particular a level of feedback between the seed coat and the endosperm/embryo. Transporters localized at the embryo surface seem to be regulated by the metabolite concentrations present in the seed apoplast, but it is unclear how these transporters contribute to coordinating carbon partitioning between the maternal and filial tissues of the seed. For example, storage protein synthesis in the *A. thaliana* embryo and final seed weight depend on nitrogen availability and are mediated by AAP1, which is expressed in both the embryo and the seed coat. In both the seed coat and the endosperm of the *aap1* loss-of-function mutant, amino acid levels are higher than in the wild type, whereas in the embryo, the content of storage proteins and carbohydrate is lower (Sanders et al., 2009). Similarly, phloem amino acid concentrations regulate nitrogen loading into the oilseed rape seed (Lohaus and Moellers, 2000; Tilsner et al., 2005; Tegeder, 2014).

Nutrient release from the seed coat needs to be precisely tuned via a fast and sensitive mechanism such as, for example, cell turgor, which directly depends on the activity of vacuolar sucrose transporters (Walker et al., 2000). A turgor-homeostatic mechanism in the seed coat could sense a loss of solute from the seed apoplast and then could act to balance this by adjusting eﬄux activity (Patrick and Oﬄer, 2001). The growth of the endosperm, therefore, may trigger a feedback signal to the seed coat, which is then transmitted via a calcium signaling cascade (Zhang et al., 2007) to drive cell elongation. Such a mechanism could allow the endosperm to coordinate aspects of seed development (Borisjuk et al., 2002; Melkus et al., 2009).

Sucrose, hexoses, and amino acids can all provide a regulatory signal (Koch, 2004; Weber et al., 2005; Ruan et al., 2010). Sugar responsiveness is a prominent feature of genes contributing to the sink strength of developing organs, and provides an important mechanism for sink adjustment to source delivery (Xiong et al., 2013b). A number of genes involved in sucrose metabolism are up-regulated by sugars (Smidansky et al., 2002; Kang et al., 2009). As a result, the strongest sink is the one most efficiently up-regulated by the supply of assimilate (Bihmidine et al., 2013). A balancing via down-regulation is also feasible (Kang et al., 2009). An invertase prominent in regulating sucrose unloading (Cheng et al., 1996; Weber et al., 2005; Chourey et al., 2011) has been proposed to enhance sugar signaling in the context of establishing assimilate sinks (Weber et al., 1996a, 2005; Ruan et al., 2010; Aoki et al., 2012). The expression of the myb-like transcription factor *ZmMRP-1*, a key regulator of transfer cell differentiation (Gómez et al., 2009), is modulated by various carbohydrates, with glucose being the most effective inducer (Barrero et al., 2009). ZmMRP1 transcriptionally activates a number of transfer cell-specific genes in the maize endosperm (Gómez et al., 2002); one of these is *Meg1*, which encodes a small cysteine-rich peptide localizing to the plasma membrane of differentiating endosperm transfer cells, where it regulates the expression of *cell wall invertase 2* (Costa et al., 2012). The strong maternal influence over placental-like functions is conferred by genomic imprinting, which has been attributed to maternal–filial co-adaptation (Wolf and Hager, 2006; Gehring et al., 2009). *Meg1* is one of more than a hundred imprinted genes active in the endosperm (Raissig et al., 2011; Waters et al., 2011; Wolff et al., 2011; Zhang et al., 2011), and is the first to have been identified as having a role in regulating the flow of nutrients to the embryo (Costa et al., 2012). *Meg1* also acts in tripartite (seed coat-endosperm-embryo) interaction and regulates maternal nutrient uptake, sucrose partitioning, and seed weight.

## MAINTAINING A LEVEL OF CONTROL OVER SEED SIZE

The developing maternal tissue has an effect on endosperm filling and thus also final seed size. Several genes associated with seed size in *A. thaliana* are expressed in the seed coat (Haughn and Chaudhury, 2005; Roszak and Köhler, 2011). Among them are the transcription factors *ARF2/MNT* [which restricts seed size by suppressing cell proliferation in the integuments (Schruff et al., 2006; Li et al., 2008)], *AP2* (Jofuku et al., 2005; Ohto et al., 2005, 2009), *TTG2* and *EOD3/CYP78A6*, which control cell expansion in the integuments (Garcia et al., 2005; Ohto et al., 2009; Fang et al., 2012) and *KLUH/CYTOCHROME P450 78A5*, which stimulates cell proliferation in the endothelium. The up-regulation of *KLUH* increases seed size, produces larger seedlings and increases seed oil content (Adamski et al., 2009). *NARS1* and *NARS2* are expressed in the outer integument, acting redundantly to regulate seed shape and embryogenesis (Kunieda et al., 2008); the seed of the *nars1 nars2* double mutant are abnormally shaped.

At least 400 quantitative trait loci (QTL) related to grain size have been identified in rice and candidate genes have been identified for some of these (Huang et al., 2013). *Dwarf1* is strongly transcribed in the early developing pericarp and only weakly in the endosperm (Izawa et al., 2010). This gene encodes the α subunit of the heterotrimeric G protein (Ashikari et al., 1999; Fujisawa et al., 1999), which is suspected of controlling cell number, since its loss-of-function mutant displays a ubiquitous reduction in cell number (Izawa et al., 2010). A second candidate gene, *gif1*, encodes a cell wall invertase required for carbon partitioning during early grain filling (Wang et al., 2008). Its transcript is only detectable in the (maternal) vascular tissue, suggesting that its role is associated with sucrose unloading (Wang et al., 2008). The expansion of parenchymatous cell layers seen in a faba bean accession (large- versus small-seeded) may reflect the activity of cell wall invertase 1 (Weber et al., 1996a).

Several factors involved in ubiquitin-related activity have been shown to influence seed size (Li and Li, 2014). In rice, a ubiquitous RING-type protein displaying E3 ubiquitin ligase activity (encoded by *GW2*) negatively regulates grain size by restricting cell division. Its loss-of-function mutant forms an enlarged spikelet hull, which allows for a greater contact area between the endosperm and the seed coat (Song et al., 2007). An uncharacterized protein, encoded by a candidate gene for a QTL for seed width mapping to chromosome 5, interacts with polyubiquitin, and acts to limit grain size, possibly by its involvement in the ubiquitin-proteasome pathway (Weng et al., 2008). However, because ubiquitin-related genes are so widely expressed – including within the endosperm – it is unclear whether their function is exclusively under maternal control. The *A. thaliana* gene *DA2* is a homolog of *GW2*; it acts in the maternal tissue to restrict the growth of the seed. The *da1* mutant produced larger and heavier seeds then wild type (“da” means “large” in Chinese; Li et al., 2008). The growth-restricting factor DA1 is an ubiquitin receptor which determines final seed size by restricting the period of integument cell proliferation (Li et al., 2008; Xia et al., 2013). The gene underlying a major grain length QTL in rice encodes a putative phosphatase 2A-type protein harboring a Kelch-like repeat domain. Its effect is manifested by inducing a higher cell density on the outer surface of the glumes and the ovary (Zhang et al., 2012).

The influence of the maternal tissue on caryopsis size has been well documented in cereals, although quite how this is achieved at the molecular and metabolic level remains unresolved (Huang et al., 2013). The extensive synteny and conserved gene structure among cereals has allowed much of the knowledge gained from rice to be exploited in crops such as maize, wheat, and barley. In particular, orthologs of *GW2* have been identified in both wheat (Su et al., 2011) and maize (Li et al., 2010).

## SMALL PROVISIONS FOR GOOD REASON

**FIGURE 5:**
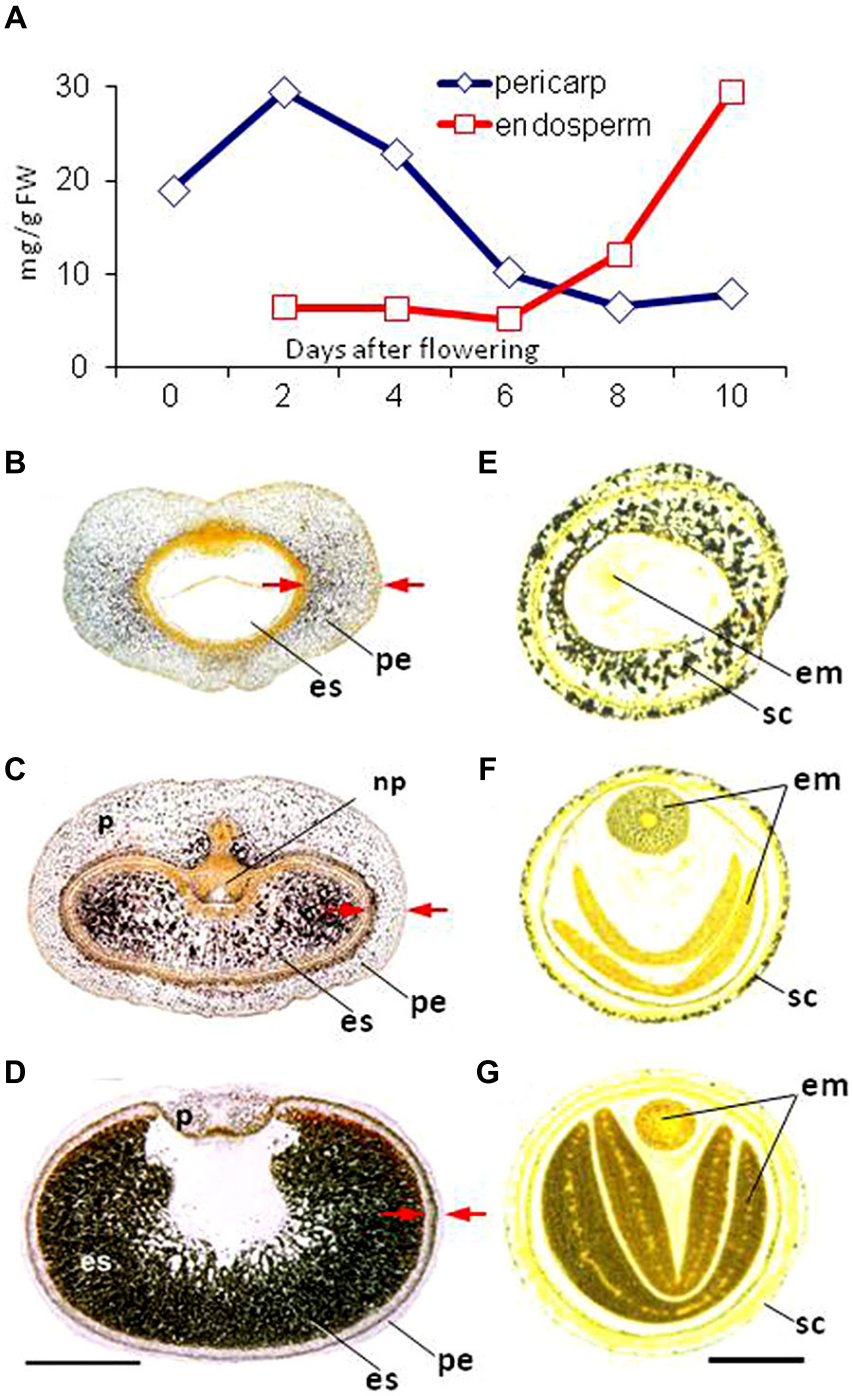
**Transient starch accumulation in the maternal tissues of the barley caryopsis and the oilseed rape seed. (A)** Transient starch accumulation (visualized in black by iodine staining) in the pericarp of the developing barley caryopsis at **(B)** 2, **(C)** 8, and **(D)** 14 days after fertilization. The pericarp is indicated by arrows. For details see Radchuk et al. (2009). **(E–G)** Starch in the seed coat of young and maturing seeds of oilseed rape. For details see Borisjuk et al. (2013). em, embryo; es, endosperm; np, nucellar projection; pe, pericarp; sc, seed coat.

At an early developmental stage, the seed coat in dicots, and the pericarp in monocots accumulate a significant amount of starch (**Figure 5**). In *A. thaliana*, oilseed rape, and pea, starch accumulation occurs in the cells of the outer integument during the growth phase (Abirached-Darmency et al., 2005; Haughn and Chaudhury, 2005; Borisjuk et al., 2013). In the *A. thaliana* seed coat, the most abundant transcript level of the genes encoding starch synthesis enzymes are observed during the pre-globular and globular developmental stages (Khan et al., 2014). Starch granules in the chalazal part of the seed coat are smaller and less abundant than in the distal part, a trait which is mirrored by a differential starch synthesizing enzyme transcript profile (Khan et al., 2014). In *M. truncatula*, an abundance of starch granules accumulates transiently in the seed coat from the embryo heart stage all the way up to mid-maturation (Verdier et al., 2013). Based on the behavior of pea seeds lacking ADP-Glc pyrophosphorylase activity, it appears that transiently produced starch is required for sink acquisition, maximal embryo growth and final seed size (Rochat et al., 1995; Vigeolas et al., 2004). The role of this transient starch is not completely clear. Young maternal tissue may perhaps have evoloved a starch storage function to ensure a sufficient assimilate sink strength, which becomes redundant once the growing seed’s own sink is established (Radchuk et al., 2009). Accumulating assimilate in the form of starch is energetically highly efficient (Schwender, 2008), so it is unsurprising that the transient maternal sink accumulates starch rather than protein or lipid.

Transient starch is utilized for the growth and development of the maternal tissue (Xiong et al., 2013a), for example, by providing a source of carbohydrate to reinforce the cell wall with pectinaceous mucilage (Khan et al., 2014). It may also help support the growth of the endosperm and embryo (Radchuk et al., 2009; Verdier et al., 2013). In *Zingiberales* and *Caryophyllales* species, the nucellus, rather than degenerating, accumulates large amounts of starch (López-Fernández and Maldonado, 2013), forming a so-called perisperm which persists until the seed is mature. In quinoa and the grain amaranth, the perisperm consists of dead, thin-walled cells completely filled with starch granules, producing a structure which strongly resembles the cereal endosperm (López-Fernández and Maldonado, 2013).

The synthesis of transient starch is performed by a similar set of genes as is used in the leaf (Radchuk et al., 2009). Its mode of breakdown in the cereal pericarp is distinct between living and dying cells. In living chlorenchyma cells, it most likely occurs via a pathway similar to that followed in the photosynthesizing leaf; this involves the phosphorylation of the starch granule surface, making it accessible for the degrading enzyme β-amylase. Plastid-localized BAM5, BAM6, and BAM7 β-amylases are thought to produce maltose, acting either at the granule surface or on linear malto-oligosaccharides. The action of iso-amylase 3 on the granule, on the other hand, releases soluble malto-oligosaccharides, which can be metabolized by disproportionating enzyme 1 (DPE1), liberating glucose, and larger malto-oligosaccharides for continued degradation. After its transport to the cytosol, maltose can be further converted to glucose by DPE2. In dying cells, the mode of starch breakdown resembles that occuring in the germinating grain, which requires a combination of α- and β-amylase activity. AMY1 is active in the pericarp and nucellar tissue of a developing grain and is responsible for most of the α-amylase activity seen in germinating grains (Radchuk et al., 2009). A plausible starch degradation pathway in dying pericarp cells involves the joint activity of AMY1 and AMY4. Linear malto-oligosaccharides released by the action of these enzymes should provide an appropriate substrate for β-amylase 2. The molecular identity of the gene/enzyme responsible for the conversion of maltose to glucose remains to be identified.

Seed coat tissues may also serve as a transient depot for proteins and microelements. In faba bean, the storage protein legumin B is deposited in the seed coat at mid-embryogenesis (Panitz et al., 1995), while the wheat pericarp and nucellus accumulate significant quantities of calcium, copper, iron, molybdenum, magnesium, manganese, and phosphorus (Wu et al., 2013; Xiong et al., 2013a). The physiological significance of this accumulation has not yet been elucidated.

## DYING QUIETLY

Because cell division in the maternal tissue ceases soon after fertilization, further enlargement only occurs through cell expansion (Radchuk et al., 2011; Figueiredo and Köhler, 2014). The rapid growth of the endosperm and embryo requires the triggering of PCD to remove maternal cells in order to allow seed expansion. In both dicotyledonous and monocotyledonous seeds, the early stages of endosperm expansion are at the expense of the nucellus (Domínguez et al., 2001; Greenwood et al., 2005; Lombardi et al., 2007; Zhou et al., 2009; Radchuk et al., 2011). After the cereal nucellus has degenerated, the next tissue to undergo PCD is the pericarp, starting from its innermost cell layer (Radchuk et al., 2011). The chlorenchyma is retained in a viable, functional state almost up to physiological maturity. In *A. thaliana* and the castor oil plant, PCD occurs first in the endosperm and later in the integuments (Greenwood et al., 2005; Nakaune et al., 2005).

As in animal cells, the molecular basis for PCD in plants relies on caspase-like activity. Although no caspase homologs have been identified in plants, plants do harbor proteases sharing some similarity to animal caspases. Caspase-1-like, caspase-3-like, and caspase-6-like activities have all been detected in the degenerating chayote (*Sechium edule*) nucellus (Lombardi et al., 2007). Vacuolar processing enzyme (VPE, also referred to as legumain) has caspase-1-like activities (Hara-Nishimura et al., 2005), while phytaspase possesses caspase-6-like activity (Chichkova et al., 2010). A seed specific δVPE produced by *A. thaliana* is present in the two inner cell layers of the seed coat. In a mutant defective for *δVPE*, PCD is delayed and the seed coat remains thick throughout development. In contrast, in the wild type, the two layers undergo PCD very early during seed development, reducing their thickness by more than 50% (Nakaune et al., 2005). The spatial and temporal patterns of *HvVPE4* transcription coincides with the onset of PCD in the barley pericarp (Radchuk et al., 2011). The product of *HvVPE2a* (also called nucellain), together with those of *HvVPE2b*, *HvVPE2c*, and *HvVPE2d*, are important for the timely degeneration of the nucellus and the nucellar projection (Linnestad et al., 1998; Radchuk et al., 2011). HvVPE2b possesses caspase-1 like activity (Julián et al., 2013). The supposed role of the barley VPEs in grain development still requires experimental confirmation. Novel technologies (Tsiatsiani et al., 2012) might help to identify the target(s) of VPE.

A large number of proteases are present in degenerating maternal tissue, some of which may be active components of PCD (Sreenivasulu et al., 2006; Thiel et al., 2008). The ricinosome, a castor oil plant specific organelle, contains a large quantity of a pro-cysteine endopeptidase (CysEP), which serves to disintegrate the nucellar cells, leaving crushed and folded cell wall residues in the apoplastic space (Greenwood et al., 2005). Nuclear DNA fragmentation has been detected in the nucellus of the castor oil plant (Greenwood et al., 2005), chayote (Lombardi et al., 2007), barley (Linnestad et al., 1998; Radchuk et al., 2011) and wheat (Domínguez et al., 2001), as well as in the *A. thaliana* endothelium (Nakaune et al., 2005).

The transcription factor *OsMADS29* has been described as a regulator of PCD in the rice nucellus (Yang et al., 2012; Yin and Xue, 2012). *OsMADS29* transcripts are concentrated in the nucellus and the nucellar projection (Yin and Xue, 2012), but are also detectable in the inner layers of the pericarp, in other maternal seed tissues and in the embryo (Yang et al., 2012; Nayar et al., 2013). PCD is slowed in an *OsMADS29* knockdown line, leading to a reduction in starch accumulation in the endosperm and the production of shrunken or aborted grain (Yin and Xue, 2012). An alternative role for this transcription factor – in relation to hormone homeostasis, plastid biogenesis and starch synthesis – has been suggested by Nayar et al. (2013). OsMADS29 is thought to bind to the promoters of cysteine protease genes (Yin and Xue, 2012). The down-regulation of *OsMADS29* suppresses the transcription of *VPE* genes in the grain (Yang et al., 2012). As yet, however, there is no consensus regarding the localization of either its transcript or its gene product, its primary target(s) or its likely function during grain development. The transcription of its barley homolog, *HvMADS29*, is restricted to the nucellus and the nucellar projection and coincides with that of *Jekyll* and *HvVPE2a*. The promoter regions of *Jekyll*, *HvVPE2a*, *HvVPE2b*, *and HvVPE2d* contain the same CArG-like regions recognized by OsMADS29, which implies that they are all transcriptionally regulated by HvMADS29. Jekyll is a key player in grain development (Radchuk et al., 2006), and is also active in nurse tissues, where it mediates the gametophyte-sporophyte interaction in both the gynoecium and the androecium (Radchuk et al., 2012). Its down-regulation slows PCD in the nucellus and nucellar projection, although the mechanistic basis of this effect is unclear. The *Jekyll* product, which is unique to *Pooideae*, is a small, cysteine-rich protein deposited within the intracellular membranes (Radchuk et al., 2012). It has no significant similarity to other proteases or any protein of known function, and has no *in vitro* protease activity.

## IMPROVING THE CHANCES OF SEED SURVIVAL

The capacity of the seed coat to limit water loss and to protect against mechanical damage persists beyond seed maturation. The mechanical strength of the seed coat is achieved primarily by the accumulation of sclerenchyma. Cell walls containing lignin, cellulose, and sometimes silica are effective in providing protection against attacks by fungi, insects, and herbivores (Lanning and Eleuterius, 1992). Seed coat constituents (e.g., PAs in *Brassica napus*) impair the digestibility and are being targeted by genetic and molecular approaches to improve nutritional value of seeds (Auger et al., 2010; Yu, 2013).

The seed coat can also contribute to seed dispersion: some species produce winged seed, where the wing structure is formed by outgrowths of the seed coat, while others produce hairy seed. Some of the compounds found on the seed coat have industrial applications. Under natural conditions, the cotton boll (its fibers are almost pure cellulose) will tend to increase the dispersion of the seeds, and indeed the use of cotton for fabric is known to date to prehistoric times. Genetically modified cotton has increased yield, but further improvements are needed (Molina et al., 2008; Kathage and Qaim, 2012; Ruan, 2013).

The seed coat, along with the endosperm, is the primary determinant of seed dormancy (Debeaujon et al., 2000; Bethke et al., 2007; Iglesias-Fernández et al., 2007), which represents a physiological adaptation to environmental uncertainty (Bewley et al., 2013). Dormancy dictates the environmental conditions required to trigger germination (Finch-Savage and Leubner-Metzger, 2006). The acquisition of seed dormancy is under complex genetic control, but it is vital as a means of assuring the survival of natural plant populations (Penfield and King, 2009; Graeber et al., 2012). Seed dormancy can be a critical trait for breeders (Fuller and Allaby, 2009; Gao et al., 2013; Smýkal et al., 2014) and would represent a prime target for biotechnology intervention, provided that its regulation were better understood (Flintham, 2000; Graeber et al., 2012).

A combination of external factors, such as light, temperature, water, and chemicals play an important role in breaking seed dormancy. Many small seeded species, the seeds of which contain little stored energy, are triggered to germinate by exposure to light. In some cases the minimum duration of exposure required can be measured in milliseconds (Quail, 1997). Phytochromes represent the main vehicle for seeds to sense light (Smith, 2000; Mochizuki et al., 2009). The linkage between the light-regulated trigger and the hormone-mediated induction of germination in *A. thaliana* has been been explored by Cho et al. (2012). A rapid adaptation to light fluctuation can represent a key competitive advantage in natural plant populations. A particularly striking example of how the seed coat contributes to seed survival is provided by species that have adapted to bushfires. Australian Banksia species are destroyed when burnt, but the fire stimulates the opening of their seed-bearing follicles and promotes the germination of buried seed. The smoke from bushfires contains as many as 5000 different compounds (Nelson et al., 2012), including a number of substances proven to stimulate germination (Nelson et al., 2012). While volatiles such as ethylene and nitric oxide are not very persistent in the soil, other smoke compounds (in particular, karrikins, and cyanohydrins) are quite stable in the upper layers of the soil where dormant seeds tend to be found. The seed coat of a mature *Banksia sp*. seed is permeable to these molecules. The seeds of hundreds of plant species have been tested with many smoke compounds, and the mode of action of some has been elucidated in *A. thaliana* (Nelson et al., 2010; Flematti et al., 2013). It has even been suggested that some of these compounds have been co-opted through evolution as signals for germination (Nelson et al., 2012; Challis et al., 2013).

## CONCLUDING REMARKS

During seed development, the seed coat (dicots) and pericarp (monocots) serve a number of functions, most of which have evolved to protect the seed and to promote the development of the embryo and the endosperm within it. The architecture, chemical composition and metabolism of the seed coat work together to ensure effective responses to both biotic and abiotic factors. Nutrients passing from the mother plant to the developing embryo and endosperm must traverse the seed coat, which therefore controls seed development and seed filling. Specialized tissues have developed in a coordinated fashion on either side of the apoplast to direct and facilitate nutrient flow toward the growing embryo and endosperm. The seed coat and the endosperm act together to determine final seed size. The fine-tuning of nutrient flow from the seed coat to their endosperm and embryo is controlled at the genetic, epigenetic, and metabolic level, but how the interplay is achieved *in vivo* remains to be clarified.

Photosynthesis in the seed coat provides oxygen to the hypoxic regions deep within the developing seed. The overwhelming proportion of the nutrition supplied to the seed is provided by the leaf of the mother plant, the delivery of which is tied to the circadian rhythm. Adapting the seed’s metabolism to this uneven flow of nutrition is facilitated by the seed’s ability to sense light. In the course of seed development, the maternal tissues undergo PCD, thereby providing both the space and nutrients for the growth of the filial tissue. Finally, an outer seed envelope is built which is important for providing protection for the mature seed, enabling the establishment of dormancy and aiding in seed dispersal.

## Conflict of Interest Statement

The authors declare that the research was conducted in the absence of any commercial or financial relationships that could be construed as a potential conflict of interest.
